# The CT Classification of Multilevel Cervical Ossification of the Posterior Longitudinal Ligament to Guide Hybrid Anterior Controllable Antedisplacement and Fusion vs. Posterior Laminoplasty

**DOI:** 10.1111/os.14088

**Published:** 2024-05-21

**Authors:** Shunmin Wang, Haibo Song, Ximing Xu, Shiyong Ling, Yuan Wang, Jingchuan Sun, Jiangang Shi

**Affiliations:** ^1^ Department of Orthopaedics Changzheng Hospital Navy Military Medical University Shanghai People's Republic of China; ^2^ 910 Hospital of China Joint Logistics Support Force Quanzhou China; ^3^ Dongying People's Hospital Dongying City China; ^4^ Shanghai Jing'an District Zhabei Center Hospital Shanghai China

**Keywords:** Hybrid Anterior Controllable Antedisplacement and Fusion, Complications, Laminoplasty, Ossification of Posterior Longitudinal Ligament

## Abstract

**Objective:**

For precise and minimally invasive treatment of ossification of the posterior longitudinal ligament of the cervical spine, the lifting segment is minimized, anterior controllable antedisplacement and fusion (ACAF) was refined and improved. In addition, the development of appropriate surgical procedures for the ossification of each segment was rarely reported. Therefore, this study aimed to compare the efficacy and safety of hybrid anterior controlled antedisplacement fusion (Hybrid ACAF) with laminoplasty for multilevel ossification of the posterior longitudinal ligament (OPLL).

**Methods:**

Between May 2018 and May 2021, 70 patients with multilevel OPLL were divided into a hybrid ACAF group and a laminoplasty group according to surgical methods. All patients were followed up for at least 1 year. Japanese Orthopaedic Association (JOA) score and recovery rate (JOARR), (VAS, NDI) score and C2–C7 Cobb angle, the sagittal vertical axis of the neck (SVA), and complications (cerebrospinal fluid leakage, C5 paralysis, etc.) were compared between the two groups by *t* test or non‐parametric test.

**Results:**

The operation time of hybrid ACAF was longer. C5 paralysis and axial pain were more common in the laminoplasty group, while dysphagia and hoarseness were more common in the hybrid ACAF group. At the last follow‐up, the hybrid ACAF group had better recovery and maintenance of cervical lordosis and sagittal plane balance and a higher JOA score and recovery rate than the laminoplasty group.

**Conclusions:**

Hybrid ACAF can reduce the number of vertebral bodies and expand the decompression range, which is safe, effective, and tailored to local conditions. Compared with laminoplasty, hybrid ACAF is a precise alternative for patients with OPLL.

## Graphical abstract

The ossification passes through the posterior edge of the C2 vertebral body and all the C3 vertebral bodies, the center of the C4 vertebral body, the lower edge of the C5 vertebral body, the upper edge of the C6 vertebral body, and the C5/6 and C6/7 intervertebral spaces. Therefore, C2 chooses shelter, C3 and C4 choose lifting, C5/6 chooses TSD, C6/7 chooses ACDF, and HACAF removes the operable ossification, and lifts the vertebral body that retains the ossification.

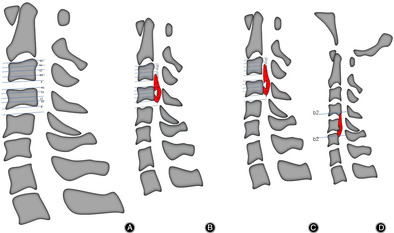

## Introduction

Ossification of the posterior longitudinal ligament (OPLL) is a degenerative disease in which ectopic ossification of the spinal ligaments leads to myelopathy or radiculopathy.^1^ Patients with moderate and multilevel severe continuous OPLL often require surgical treatment, including anterior, posterior, and combined anterior–posterior approaches.^2^, ^3^ Posterior surgery is limited by indirect decompression, and the clinical efficacy is worse than that of anterior surgery.^4^ Anterior controllable antedisplacement and fusion (ACAF) may replace the combined approach and is suitable for patients with an ossification greater than 60%.^5^, ^6^

In addition to the degree of ossification, the sagittal morphology of ossification also affects the degree of surgical difficulty and extent of decompression.^7^, ^8^, ^9^ The various OPLL classification systems have been widely researched. Most of these systems describe and classify the shape of ossification but offer little guidance in choosing surgical modalities and procedures.^8^, ^9^ The classification schemes mainly provide guidance and prognosis for patients with severe OPLL in choosing ACAF treatment. For precise and minimally invasive treatment of OPLL of the cervical spine, the lifting segment is minimized. There are fewer CT classifications for OPLL, a new classification was originally proposed to refine and improve the ACAF.

Through retrospective analysis of the follow‐up data of hybrid ACAF and laminoplasty in patients with OPLL, the following objectives were achieved: (i) to propose an original CT type for OPLL; (ii) to explore the feasibility and surgical strategy and analyze its clinical effects and imaging changes of hybrid ACAF; (iii) to compare the therapeutic effects of the two procedures.

## Materials and Methods

### Study Design

The patients enrolled in this study were required to meet the following criteria: (i) OPLL diagnosis confirmed by CT; (ii) meets the criteria for the use of the anterior approach (i.e., the ossification range ≥ 3 segments, continuous or not); (iii) myelopathy symptoms, that is, magnetic resonance imaging (MRI) scans showing compression of the cervical spinal cord, mainly due to OPLL; (iv) receiving laminoplasty technology or hybrid ACAF in our department between May 2018 and May 2021; and (v) complete continuous follow‐up for more than 1 year. The exclusion criteria were as follows: (i) myelopathy not attributable to OPLL; (ii) history of cervical spine trauma, ankylosing spondylitis, infection, or malformation; (iii) cannot tolerate surgery; and (iv) previous surgical history. The ethics committee of our institute agreed and approved the study (No. SHDC2020CR1024B). Altogether, 70 patients met the criteria. Demographic and clinical characteristics, including age, sex, duration of symptoms, smoking and alcohol consumption, and the status, type, extent, occupancy of OPLL, operating time, blood loss, postoperative hospitalization, and total complications, are revealed in Table 1.

**Table 1:** Demographic and clinical characteristics in the HACAF and laminoplasty groups.

Feature	HACAF group (*n* = 27)	Laminoplasty group (*n* = 43)	Statistical values (*t*/*x* ^2^)	*p*
Male: Female	16/11	24/19	0.080	0.777
Age (years)	62.96 ± 9.34	59.47 ± 10.60	1.405	0.164
Diabetes mellitus (yes)	11 (40.7)	17 (39.5)	0.010	0.920
History of smoking (yes)	19 (70.4)	24 (55.8)	1.483	0.223
No. of segments	2.41 ± 1.05	3.33 ± 1.06	−3.538	<0.001
Estimated blood loss (mL)	320.30 ± 147.32	419.91 ± 118.36	−3.116	0.003
Operating time (h)	250.22 ± 38.79	123.37 ± 15.86	19.112	<0.001
OR of OPLL	72.15 ± 6.87	70.51 ± 6.38	1.014	0.314
Length of postoperative hospitalization (days)	8.29 ± 1.86	7.79 ± 2.23	0.982	0.329
Total complications n (%)	5 (18.5)	11 (25.6)	0.469	0.493
Dysphagia	1	1		
Hematoma	0	0		
CSF leakage	1	1		
Neurological deterioration	0	0		
C5 plasy	0	3		
Axial pain	1	5		
Hoarseness	1	0		
Postoperative infection	1	1		

Abbreviation: BMI, Body mass index.

To reduce study bias, we screened strictly according to inclusion criteria, based on a computerized random lottery, followed standard surgical procedures, and evaluations and follow‐up were performed by third parties.

### CT Classification

**Figure 1:**
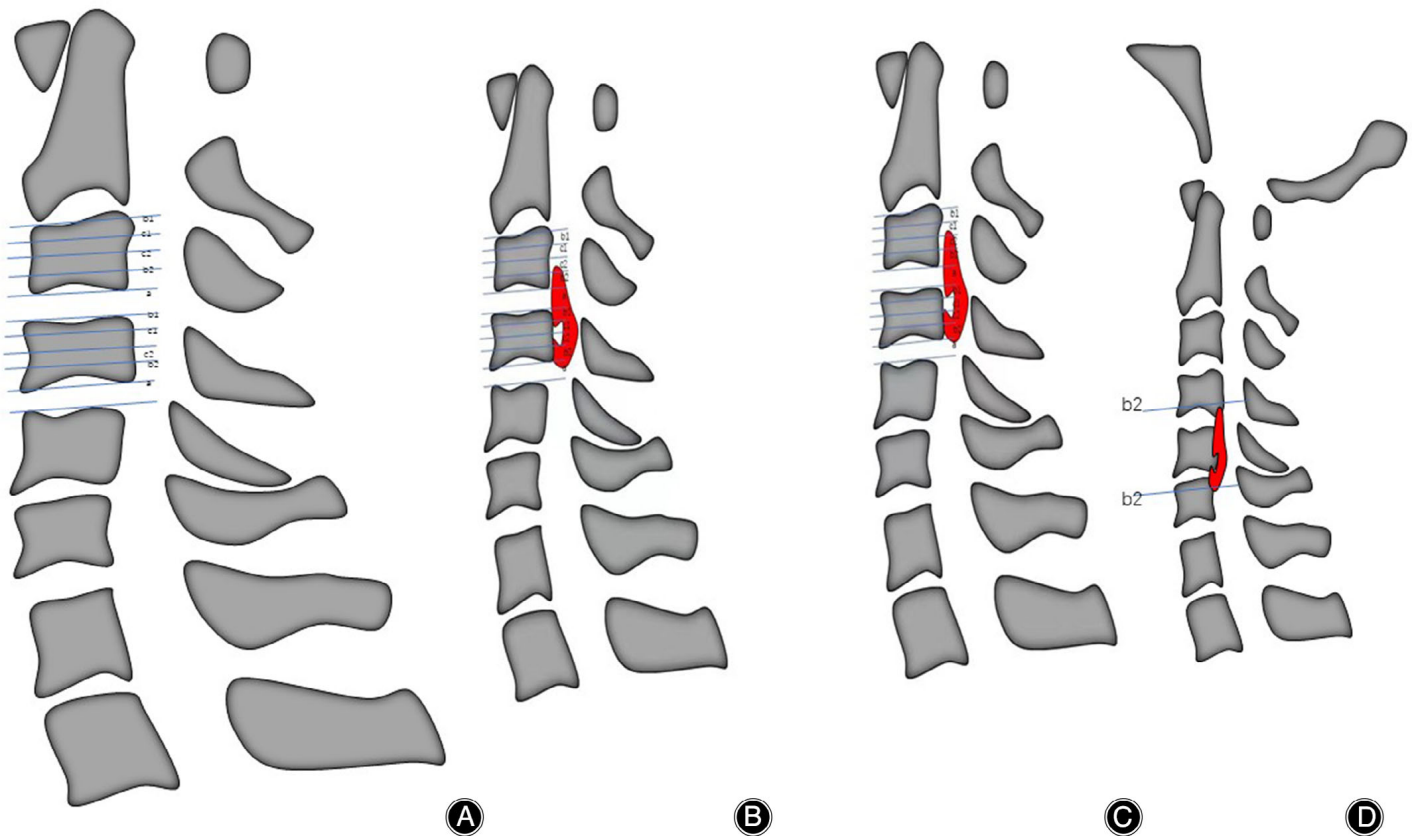
(A) sagittal classification of OPLL based on its distribution. a: noncontinuous ossified mass located behind the intervertebral space. b1: noncontinuous ossified mass located at the posterior margin of the upper 25% vertebral body. c1: noncontinuous ossified mass located at the upper middle part of the vertebral body. c2: noncontinuous ossified mass located at the lower middle part of the vertebral body. b2: noncontinuous ossified mass located at the posterior margin of the lower 25% vertebral body. The shelter technique can be used when the ossified mass does not exceed half of the vertebral body and is continuous with adjacent segments, B: shelter + 1 vertebral body ACAF. C: 2 vertebral body ACAF. D: 2 shelters + 1 vertebral body ACAF.

**Figure 2:**
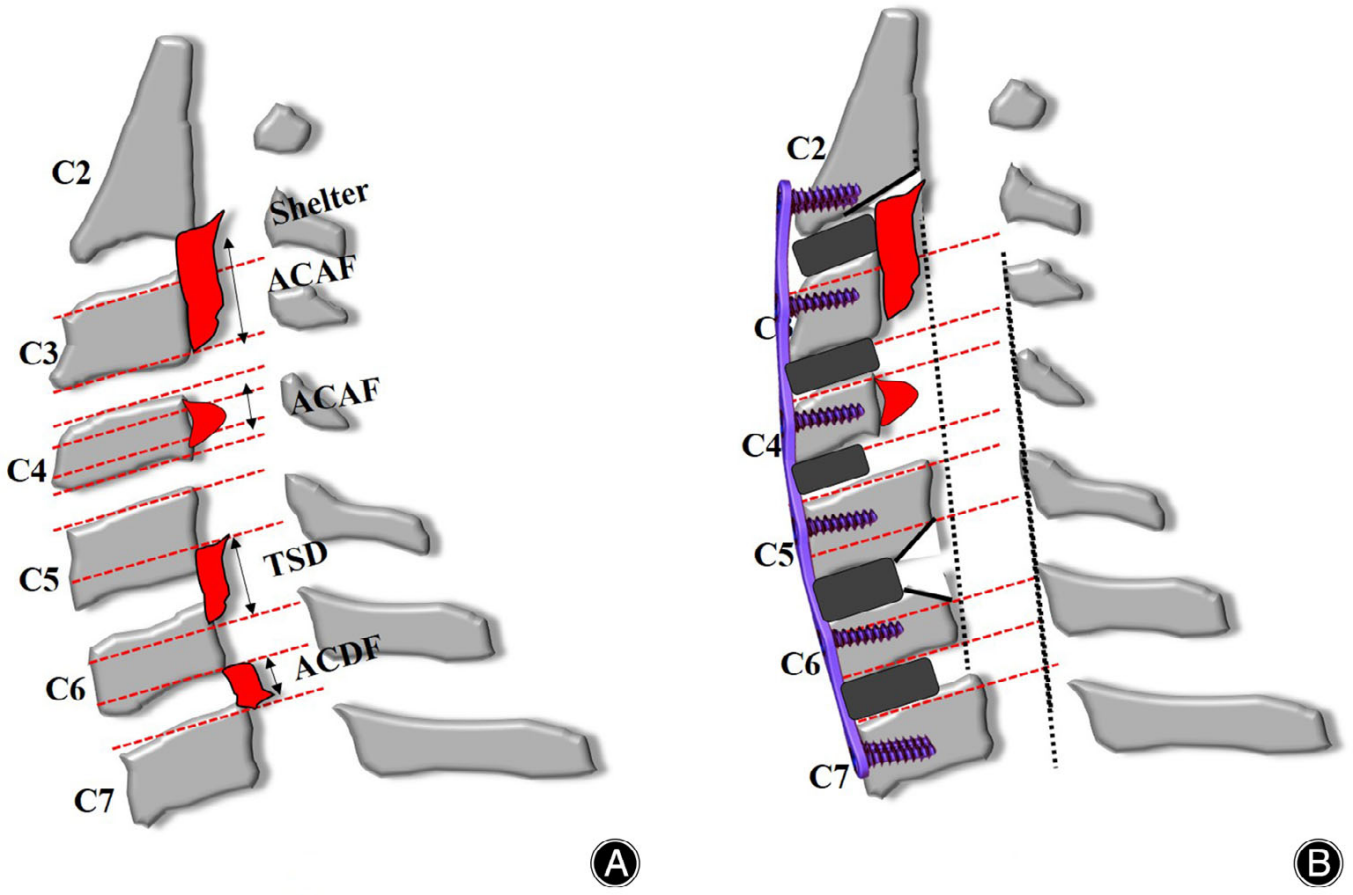
(A) The ossification passes through the posterior edge of the C2 vertebral body and all the C3 vertebral body, the center of the C4 vertebral body, the lower edge of the C5 vertebral body, the upper edge of the C6 vertebral body, and the C5/6 and C6/7 intervertebral spaces. (B) C2 chooses shelter, C3 and C4 choose ACAF, C5/6 chooses TSD, C6/7 chooses ACDF, and HACAF removes the operable ossification, and hoists the vertebral body that retains the ossification.

**Figure 3:**
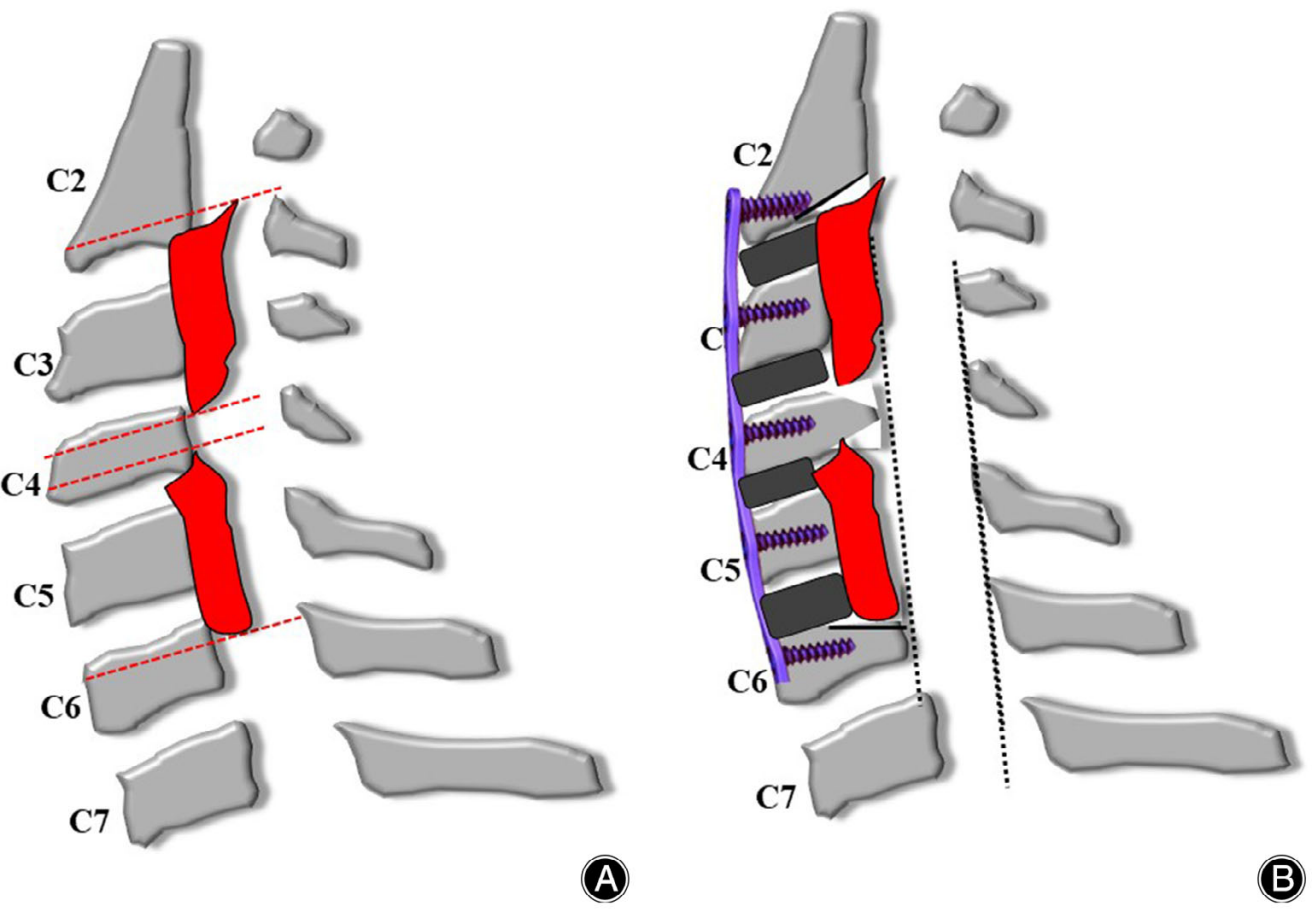
(A) The ossification passes through the lower edge of the C2 vertebral body, all the C3 and C5 vertebral bodies, and the upper and lower edges of the C4 vertebral body. (B) C2 chooses shelter and to hoist C3 and C5. C4 does not need to be hoisted and wedge decompression is chosen at this level.

The main classification divides the disc level and vertebral body into four equal sections (Figures 1, 2, 3). The central half is level C, the two sides are level B, and the intervertebral space is level A. Two spine surgeons used three stratification schemes to independently classify each segment of the mixed group OPLL. Had opinions differed, a third senior assessor made the final decision. Internal and interobserver consistency of OPLL stratification was analyzed using kappa statistics.

### Hybrid ACAF and Laminoplasty

When the ossifications causing spinal cord compression were mainly located in Level A (a), ACDF was selected to remove the ossifications and curettage the osteophytes. The shelter^10^ (b2 + c2) or TSD technology (b1 or b2)^11^ and stealth wedge decompression was chosen; for level C (c1 + c2), the hoisting vertebra was selected. Hybrid ACAF quantifies the procedure for each site of ossification (Figure 1).

**Figure 4:**
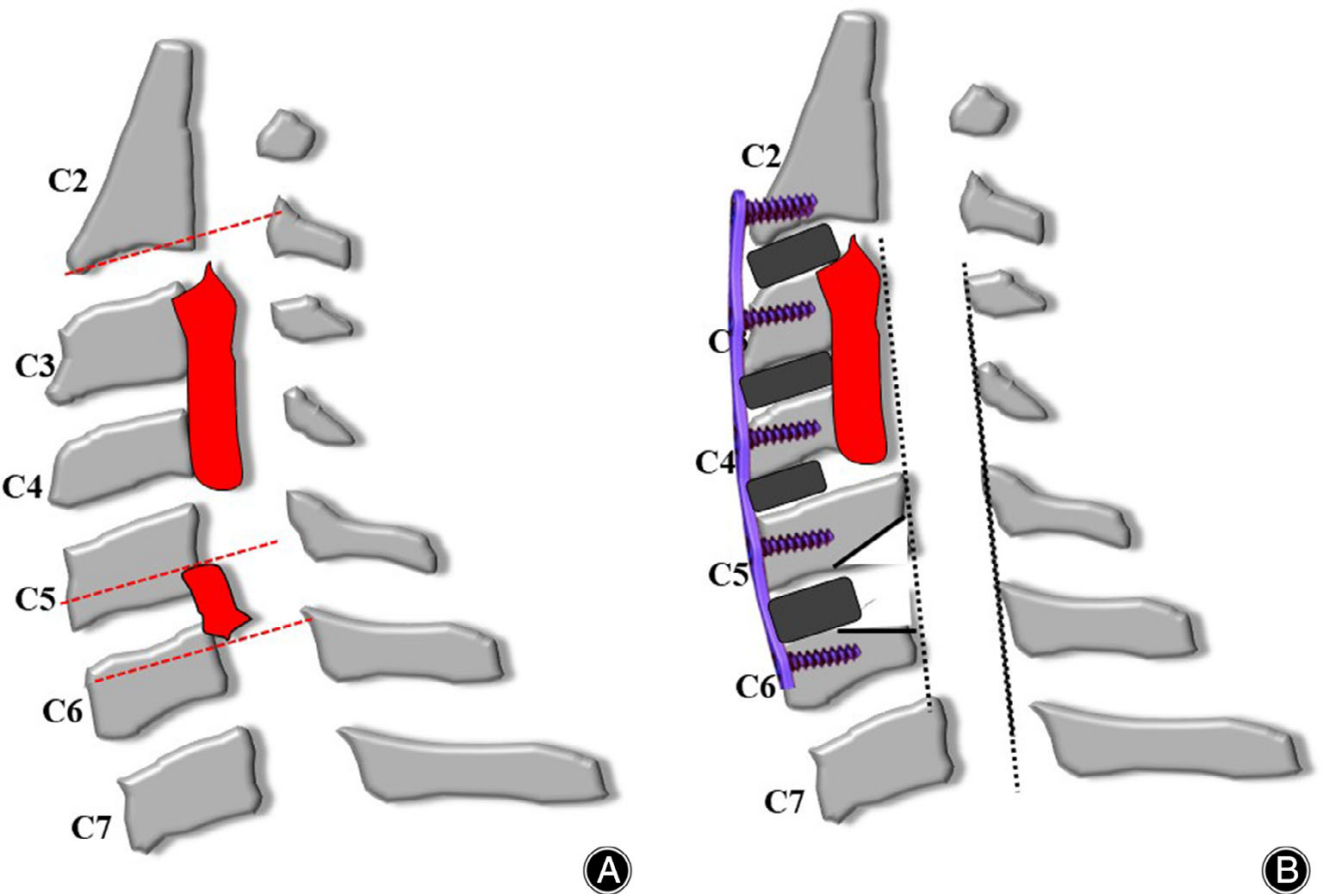
(A) The ossification passes through all C3 and C4 vertebral bodies, the lower edge of C5 vertebral body, the upper edge of C6 vertebral body and the C5/6 intervertebral space. (B) ACAF was selected in C3 and C4 level, and TSD in C5/6.

The surgical procedure for ACAF has been described in our previous study.^11^ The procedure for hybrid ACAF was as follows: (1) a standard right approach was used to expose the cervical spine, and intraoperative fluoroscopy was used to confirm the operative level. (2) Removal of the involved disc tissue: The posterior longitudinal ligaments of the head and tail of the OPLL, which required the lifting segment, and the osteopathic vertebrae of the anterior and posterior margins were appropriately scraped. Surgical procedures were selected according to stratification. (3) Removal of the anterior vertebral bone that needed to be pulled according to the thickness of ossification, suitable cages containing bone fragments of autogenous bone were placed in each intervertebral space. (4) High‐speed grinding and Kerrison Rongeur formed a 2–3‐mm wide groove on the left side of the vertebral body, and a suitable curved titanium plate was installed, and screws were inserted into the vertebral body for temporary fixation. (5) In the same way, the right groove was conducted to confirm that the vertebrae and posterior longitudinal ligament were completely isolated. Finally, the loose screws were screwed and tightened to hoist the vertebrae anteriorly to enlarge the spinal canal. Fluoroscopy revealed that the cervical spine internal fixation and fusion system was in a good position. All patients had improved symptoms after surgery and a neck brace was worn for 2–3 months (Figures 1, 2, 3, 4).

Surgical procedure for laminoplasty has been described in the literature with the ARCH micro‐plate fixation system. All patients were prescribed with a neck brace for 1 month after surgery.

### Clinical and Imaging Data

All patients or guardians were informed about both surgical procedures, including potential benefits and risks as well as complications. The Clinical Research Ethics Committee approved the protocol. The clinical data of the patients were analyzed retrospectively. Basic information collected included age, sex, duration, time of operation, and blood loss. Symptom improvement was assessed using the following indicators: the preoperative Japanese Orthopaedic Association (JOA) score, postoperative JOA score, JOA recovery rate (JOARR), and 1‐year remission (VAS, NDI) score. Camera Measure software (v2.1.3.250, e2eSoft) was used to measure imaging data, including cervical curvature and SVA. The JOARR was calculated as follows: Recovery rate (%) = (postoperative JOA – preoperative JOA)/(17 − preoperative JOA) × 100%.

The posterior neck and shoulder pain and neck dysfunction index (NDI) were evaluated using the VAS scores. All patients underwent cervical radiographs, CT, and magnetic resonance imaging (MRI) before surgery. OPLL stratification was based on the sagittal CT images. The overall survival (OR) was defined as the ratio of maximum ossification thickness to vertebral canal diameter on axial CT images. Cervical lordosis was assessed using the C2–C7 Cobb angle. The sagittal vertical axis of the neck (SVA) is defined as the distance between the vertical line of the C2 vertebral body and the posterior upper angle of C7.

The bone fusion of the patients was demonstrated by the absence of movement between spinous processes on dynamic lateral X‐ray radiographs or the presence of continuous bridging bone trabeculae at the interface of the graft endplate, as confirmed by the CT scan.^12^

Perioperative events were recorded, including operation time, intraoperative blood loss, and significant complications, such as hematoma, cerebrospinal fluid leakage, neurological deterioration, C5 paralysis, axial pain, dysphagia, and hoarseness.

### Statistical Analysis

Three experienced observers were assigned to independently evaluate the radiometric results of the patients and calculate the average value of the three measurements. SPSS 24.0 statistical software (IBM, Armonk, NY, USA) was used for the statistical analyses. Based on the sample size, the Kolmogorov–Smirnov (KS) normality test was used to evaluate the homogeneity and variance of the normal distribution. Normally distributed data were expressed as mean ± standard deviation (SD), and inter‐group tests were performed using either a *t*‐test or a corrected *t*‐test. The non‐normal distribution was tested using the Mann–Whitney *U* or rank sum test. The chi‐square test was used to examine categorical variables. A probability value of *P* < 0.05 was considered statistically significant.

## Results

### Surgical Data and Complications

Compared with laminoplasty, the hybrid ACAF group had lower average blood loss, longer average operation time, and fewer surgical segments (*p* = 0.003, <0.001, 0.001). There were no statistically significant differences in age, sex, smoking history, diabetes, and spinal canal invasion rate between the two groups (*p* > 0.05). The complication rates were similar in both groups (*p* = 0.493). There were no significant cerebrospinal fluid leakage and neurological deterioration. C5 paralysis and axial pain were more common in the laminoplasty group, while dysphagia and hoarseness were more common in the hybrid ACAF group.

The cerebrospinal fluid leakage drainage tube was removed, and a pressure dressing was applied to cure it. At the last follow‐up, dysphagia and hoarseness were gradually self‐healing, and none of the patients complained of neurological deterioration or the need for revision surgery. (Table 1).

### Clinical and Radiological Results

There were no significant differences in preoperative JOA, VAS, and NDI scores between the two groups (*p* > 0.05). At the final follow‐up (1 year after surgery), postoperative scores were significantly improved in both groups. Compared to the laminoplasty group, the hybrid ACAF group had higher RR and JOA scores, lower VAS scores (*p* < 0.05), and no significant difference in the NDI score (*p* > 0.05).

**Figure 5:**
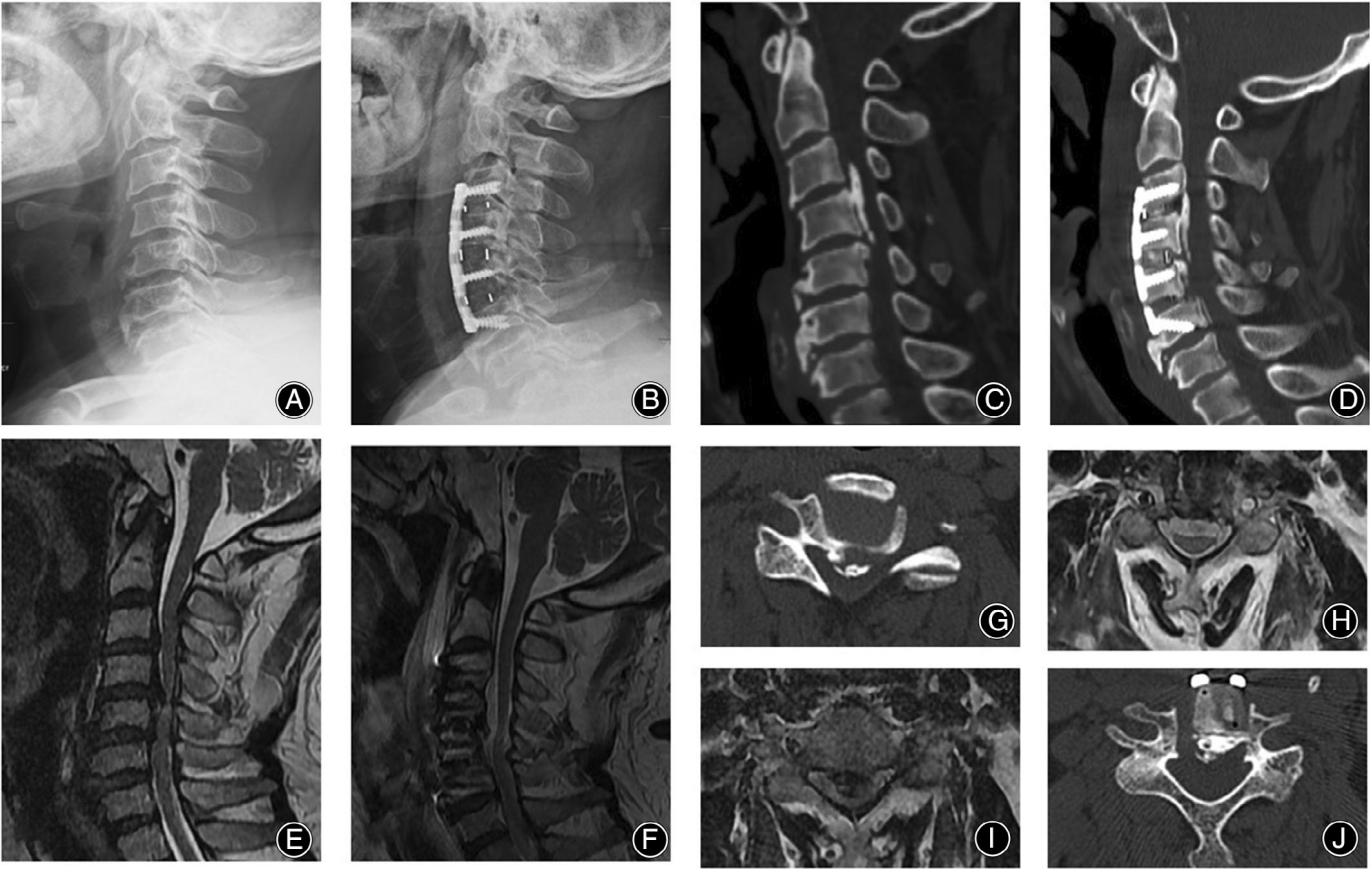
Imaging of a 63‐year‐old man undergoing HACAF. (A, C, E, J, I) Preoperative examination showed ossification of the posterior longitudinal ligament from C3 to C6, dural sac compression, and spinal cord compression and deformation. Intraoperative lifting of C4 and C5 resulted in flail decompression of the inferior border of the posterior wall of C3. During lifting, the ossification located posterior to C3 was moved anteriorly to the lower edge of the posterior wall of C3 after decompression to avoid lifting of C3, and flail decompression was performed on the upper edge of the posterior wall of C6. In addition, the ossification of C5/6 was discontinuous, so C6 was not lifted. (B, D, F, H, J). Postoperative examination showed overall anterior displacement of the vertebral body‐ossification complex, enlargement of the spinal canal, adequate decompression of the spinal cord, and the presence of cerebrospinal fluid bands.

**Figure 6:**
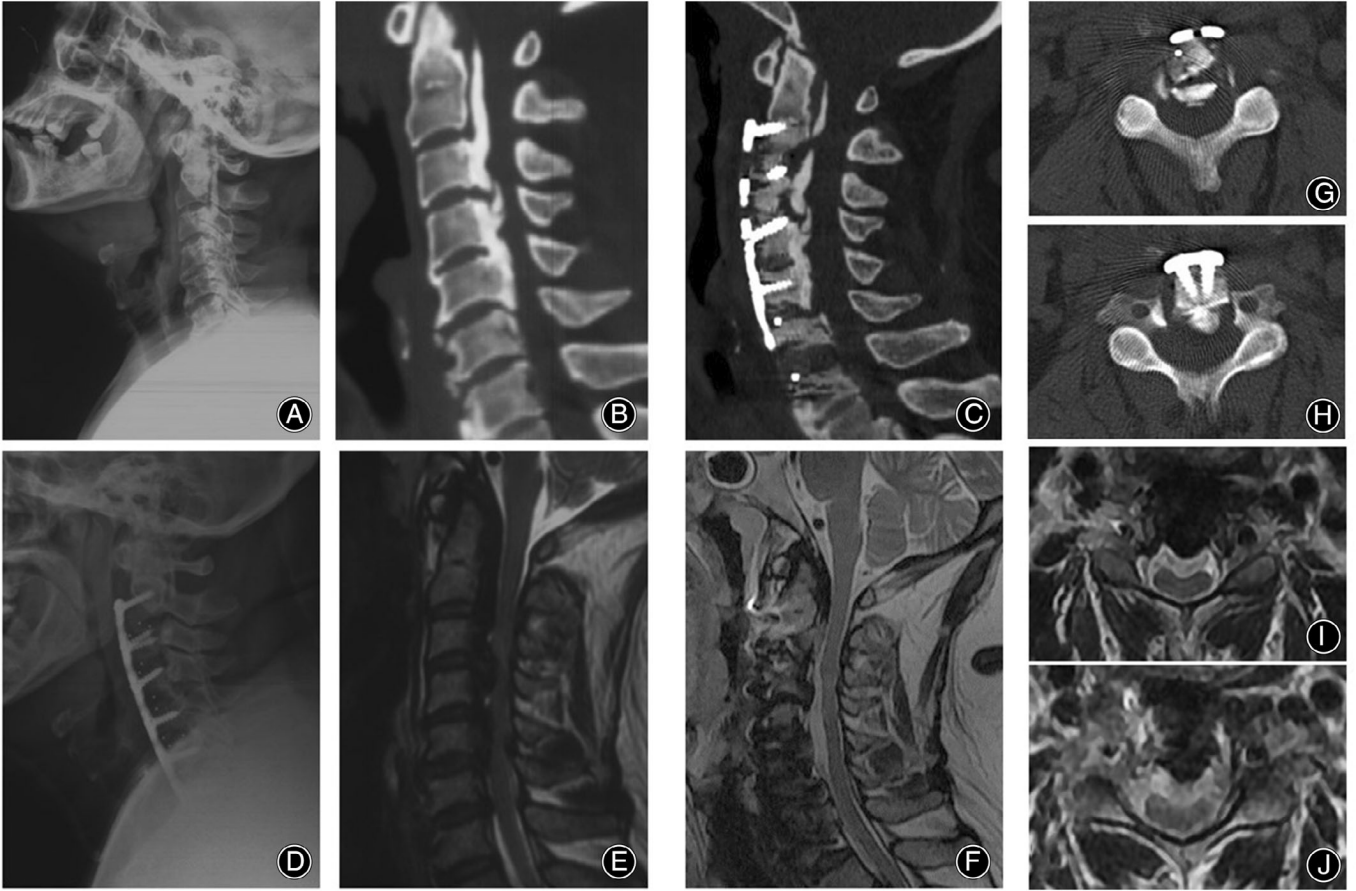
Imaging of a 54‐year‐old male undergoing HACAF. (A, B, E) Preoperative examination showed ossification of the posterior longitudinal ligaments from C2 to C5, disc herniation at C6/7, dural sac compression, spinal cord compression and deformity, and cervical stenosis. Intraoperatively, C3–C5 were lifted and the shelter technique was performed at C2 to achieve decompression of the inferior border of the posterior wall of C2, and ACDF decompression was performed at C6/7. (C, D, F–J) The postoperative period shows an overall anterior displacement of the vertebral body‐ossification complex, enlargement of the spinal canal, adequate decompression of the spinal cord, and a continuous cerebrospinal fluid band.

There was no significant difference between the preoperative baseline of C2–C7 Cobb angle and SVA in both groups. However, at the final follow‐up, hybrid ACAF improved the C2–C7 Cobb angle and SVA better than the laminoplasty (*p* < 0.05) (Table 2). According to our results, the hyperextension and hyperflexion X‐ray films of the hybrid ACAF group showed no abnormal movement between the spinous processes and intervertebral instability, and CT scan confirmed that there was continuous bridging bone trabeculae at the interface between the graft and the endplate, with no abnormal settlement found. The compatibility between the ossifications and the fusion device was high, and the fusion rate reached 100% (Figures 5 and 6).

**Table 2:** Comparison of clinical scores and radiological results between two groups.

Feature	HACAF group (*n* = 27)	Laminoplasty group (*n* = 43)	Statistical values (*t*)	*p*
JOA (score)				
Preoperative	9.48 ± 2.78	9.49 ± 1.10	−0.015	>0.05
Final	14.63 ± 1.71	13.44 ± 1.74	2.800	0.007
RR (%)	69.55 ± 22.75	51.19 ± 25.67	3.040	0.003
VAS (score)				
Preoperative	5.56 ± 1.25	5.67 ± 1.41	−0.358	>0.05
Final	1.59 ± 1.22	3.30 ± 1.85	−4.261	<0.001
NDI (score)				
Preoperative	36.59 ± 4.53	35.53 ± 3.90	1.037	>0.05
Final	12.74 ± 3.45	13.26 ± 3.23	−0.633	>0.05
C2–C7 Cobb angle				
Preoperative	9.59 ± 6.37	11.02 ± 6.71	−0.885	>0.05
Final	23.70 ± 3.21	14.28 ± 5.35	8.260	<0.001
SVA, mm				
Preoperative	25.78 ± 7.21	23.74 ± 7.40	1.130	>0.05
Final	19.56 ± 4.38	30.51 ± 5.34	−8.931	<0.001

## Discussion

Whether anterior or posterior surgery is the best treatment for OPLL remains uncertain.^2^, ^4^, ^13^ Anterior surgical excision of ossified matter, while providing direct decompression, is complicated and carries a high risk of hematoma, cerebrospinal fluid leakage, and nerve damage.^13^ Laminoplasty is relatively safe and has low technical requirements for complex and severe multilevel OPLL. However, the degree of decompression is not enough, the incidence of C5 paralysis is high, and the kyphotic changes and ossifications can be regrown after surgery.^14^, ^15^ Therefore, the combined anteroposterior approach appears to be the standard of care for these patients. However, the disadvantages include high medical costs, trauma, and long recovery time.^3^ By advancing the vertebrae‐posterior longitudinal ligament complex, ACAF directly expands the volume of the spinal canal and has a significant effect. In addition, it preserves the integrity of the spinal canal and restores the position of the spinal cord. It also avoids dealing with the dura mater and adhesions or ossifications; hence it offers safety in treating these patients.^6^, ^16^

### Main Findings

A prospective randomized trial of patients with kyphosis and high rate ossification (≥60%) treated by ACAF depicted better neurological improvement than those treated by laminoplasty.^6^ However, due to the continuous development of ACAF, determining the number of hoisted vertebrae and reducing the segments to be hoisted is an important problem to be ascertained. We found in our clinical practice that we could remove the ossification in the posterior quarter of the vertebral body with wedge decompression. The hybrid ACAF technique exposes the ossification in the posterior area of the vertebral body.^10^ It can expand the operation space and visualize the ossifications in the 25% region of the posterior edge of the vertebral body.^11^ At the same time, the lower margin of the posterior wall of the uppermost vertebral body is subject to stealth decompression during the operation. During the hoisting process, the ossifications located in the uppermost vertebral body are moved forward to the lower margin of the posterior wall of the uppermost vertebral body after decompression. Stealth decompression and lifting of the upper posterior wall edge of the lowest vertebral body can be performed simultaneously without affecting the stability of the segment. Thus, when the ossification extends to the posterior side of the vertebral body and the cephalic and caudal sides, stealthily decompression can be safely performed without pulling the segment, thus achieving adequate decompression without disrupting structural stability.^17^ Hybrid ACAF is a hybrid technique consisting mainly of ACAF and supplemented by ACDF, shelter, or TSD. It can remove the lesions behind the vertebral body; reduce the hoisting segment, trauma, and surgical fixation area; and safely expand the decompression area.

### Safety of HACAF

The incidence of CSF leakage was similar between the two groups. In addition, none of the patients experienced neurological deterioration, and hybrid ACAF was a safe surgical procedure. Early dysphagia and hoarse voice due to esophageal traction did not require specific treatment over time. In addition, the incidence of C5 paralysis and axial pain in hybrid ACAF can be significantly lower than with laminoplasty.^18^ Due to the small anterior cervical incision, less muscle, and natural intermuscular approach, there was no significant difference in the risk of postoperative infection or the mean length of hospital stay, although the mean operation time was longer for hybrid ACAF.

### Clinical Efficacy of HACAF

Although the follow‐up time was short, it showed better improvement of the JOA score RR and reduce the VAS score in HACAF group. Our imaging analysis demonstrated that hybrid ACAF was similar to ACAF. Cervical lordosis and sagittal balance were better than that in laminoplasty group. At the same time, it reduced the number of hoisted levels, leading to a better biomechanical stability, avoiding postoperative kyphosis, and maintaining the short‐term results of the surgery. In addition, it reduced the need for bilateral grooving of mobile vertebrae and had a higher fusion rate, which may be related to the osteogenic microenvironment of patients with OPLL.^19^ If the width of the osteotomy groove is too wide, the remaining vertebral body is not wide enough and the bone mass is less, which affects the screw placement. If the width of the osteotomy groove is too small and may collide with the ossification, preventing the vertebral body from moving forward. It is an important factor affecting the vertebral body hoisting and clinical efficacy in ACAF. It is necessary to measure the distance between the two edges of the bone and the vertebral artery before operation, and rationally plan the width of the groove.

### Biomechanical Stability of HACAF

In vitro, biomechanical studies revealed that the biomechanical stability of ACAF and ACDF was similar and significantly higher than that of ACCF.^13^ Immediate and long‐term biomechanical stability can be achieved after ACAF.^17^ In addition, the combination of ACDF and cervical disc replacement (CDR) surgery (HS) is biomechanically beneficial for the motion preservation of the operative segment with fewer adverse effects on adjacent segments.^20^ It resulted in fewer biomechanical and kinematic changes at the untreated level and demonstrated no additional biomechanical effects on the middle segment compared to ACDF.^21^ Compared with ACDF, three‐segment HS results in faster recovery rates and better radiological outcomes.^22^ Similar to ACAF, hybrid ACAF, combined with the mechanical characteristics of ACDF, provides better biomechanical stability and has the advantage of maintaining cervical curvature and cervical joint motion.^23^ Near the hoisting or fusion level, biomechanical changes are not limited to the intervertebral disc but can also be propagated to the rear of the vertebral body. Changes in the intervertebral disc and posterior facet joints after ACDF can be lower than those after ACCF.^24^ Therefore, there was a negative correlation between the changes in adjacent segments and number of fused and pulled vertebrae. HYBRID ACAF reduces the number of displaced vertebrae, disc pressure, and degeneration of adjacent segments. Further long‐term follow‐up studies are thus needed to determine the causes and true effects of these changes.

### Clinical Significance of HACAF

Altogether, hybrid ACAF takes advantage of various anterior surgical procedures and has a broad prospect. Several patient factors, such as OPLL level and size and the skills and surgical experience of the surgeon, should be considered when choosing this approach.^25^ In addition, high‐quality studies should be conducted to optimize the hybrid ACAF procedure for each patient with OPLL.

### CT Classification Significance

CT more adequately demonstrates the extent of the ossified OPLL, allows precise detection of the exact location, size, and shape of the ossified lesion, and has become the standard tool for evaluating the OPLL.^26^ Previous CT‐based classification of OPLL has several disadvantages^27^, ^28^: the clear definition of each type is unclear; it does not adequately convey a precise assessment of ossified lesions at each vertebral body and intervertebral level; and it cannot be tailored to guide anterior surgical treatment based on type. In axial image classification, vertebral canal occupancy and typing are very important, and for ACAF it is important to identify the lifting segments, so we propose the sagittal OPLL classification. It evaluates ossified lesions at all vertebral and intervertebral levels and allows selection of appropriate anterior surgery based on ossification at each site. It has been validated to be well suited for daily clinical use and provides new information about ossification lesions in the OPLL, which can help guide surgical decisions and data collection for future studies.

### Limitations and Strengths

There are some limitations to this study that are worth noting. The sample number was relatively small, and the follow‐up time was short. The higher incidence of C5 nerve root paralysis and axial pain in OPLL treated with laminoplasty may be related to the higher number of patients with cervical curvature loss and unilateral ossification selected in this study. Ossification without excision requires observation of its progress. In addition, cuneiform resection and decompression are required.

But importantly, this study has the following advantages: (i) precise measurement and calculation of ossifications behind each segment, minimizing the number of lifting segments; (ii) evaluates the possibility of excision of ossification in different locations to make the surgery more minimally invasive; (iii) synthesizes the advantages of each anterior surgery and adapts to local conditions to make the formulation of surgical strategies more accurate.

## Conclusion

Studies have revealed that hybrid ACAF is a precision treatment for each level of ossification. Compared with laminoplasty, hybrid ACAF, combined with the many advantages of anterior surgery, achieves better clinical efficacy.

## Funding

The study is supported by the National Natural Science Foundation of China (Grant/Award Numbers: No. 81802218), Major Special Project of Three‐year Action Plan to Promote Clinical Skills and Clinical Innovation Ability of Municipal Hospitals, Shanghai Hospital Development Centre (Grant No. SHDC2020CR1024B), Scientific Research Program of Shanghai Municipal Health Commission (Grant No. 202140318) and Shanghai Jing'an District Health Research Project (Grant No. 2021MS09).

## Conflict of Interest Statement

The authors declare that they have no competing interests.

## Ethics Approval and Consent to Participate

All procedures performed in studies involving human participants were in accordance with the ethical standards of the Ethics Committee of Changzheng Hospital Ethics Committee of Changzheng and 910th Hospital.

## Consent for Publication

Informed consent was obtained from all individual participants included in the study.

## Author Contributions

Shunmin Wang has contributed to the conception, study design, and was a major contributor in drafting of the study and writing the manuscript. Haibo Song, Ximing Xu, Shiyong Ling, and Yuan Wang have contributed to the acquisition, analysis, and interpretation of the data. Jingchuan Sun and Jiangang Shi finished the final assessment of the manuscript. All authors read and approved the final manuscript.

## Data Availability

Data will be made available on request.
